# Ketamine decreases inflammatory and immune pathways after transient hypoxia in late gestation fetal cerebral cortex

**DOI:** 10.14814/phy2.12741

**Published:** 2016-03-31

**Authors:** Eileen I. Chang, Miguel A. Zárate, Maria B. Rabaglino, Elaine M. Richards, Thomas J. Arndt, Maureen Keller‐Wood, Charles E. Wood

**Affiliations:** ^1^ Department of Physiology and Functional GenomicsUniversity of Florida College of MedicineGainesvilleFlorida; ^2^ CEPROCORNational Scientific and Technical Research Council (CONICET)CórdobaArgentina; ^3^ Department of PharmacodynamicsUniversity of Florida College of PharmacyGainesvilleFlorida

**Keywords:** Fetal hypoxia, frontal cortex, immune response, ketamine

## Abstract

Transient hypoxia in pregnancy stimulates a physiological reflex response that redistributes blood flow and defends oxygen delivery to the fetal brain. We designed the present experiment to test the hypotheses that transient hypoxia produces damage of the cerebral cortex and that ketamine, an antagonist of NMDA receptors and a known anti‐inflammatory agent, reduces the damage. Late gestation, chronically catheterized fetal sheep were subjected to a 30‐min period of ventilatory hypoxia that decreased fetal PaO_2_ from 17 ± 1 to 10 ± 1 mmHg, or normoxia (PaO_2_ 17 ± 1 mmHg), with or without pretreatment (10 min before hypoxia/normoxia) with ketamine (3 mg/kg, i.v.). One day (24 h) after hypoxia/normoxia, fetal cerebral cortex was removed and mRNA extracted for transcriptomics and systems biology analysis (*n* = 3–5 per group). Hypoxia stimulated a transcriptomic response consistent with a reduction in cellular metabolism and an increase in inflammation. Ketamine pretreatment reduced both of these responses. The inflammation response modeled with transcriptomic systems biology was validated by immunohistochemistry and showed increased abundance of microglia/macrophages after hypoxia in the cerebral cortical tissue that ketamine significantly reduced. We conclude that transient hypoxia produces inflammation of the fetal cerebral cortex and that ketamine, in a standard clinical dose, reduces the inflammation response.

## Introduction

Physiological responses to transient hypoxia are thought to protect the fetal brain and heart from the damaging effects of temporary interruptions in oxygen delivery. Cardiovascular and neuroendocrine reflex responses redirect the fetal combined ventricular output and increases blood flow toward brain, heart, and adrenals and away from skeletal muscle (Cohn et al. 1974). Severe or prolonged hypoxia produces multiple organ damage as a result of oxygen deprivation and metabolic acidosis. Cerebral cortex, cerebellum, and hippocampus are more susceptible to a hypoxic stimulus compared to other brain regions (Choi and Rothman 1990).

There have been several reports of increased markers of brain inflammation after ischemia or asphyxia in utero (Yafeng et al. 2009; Guo et al. 2010). The fetal inflammatory response is characterized by an increase in proinflammatory mediators such as IL6, IL1*β*, and TNF*α* expressed in activated immune cells in hypoxic brain regions (Rees and Inder 2005). Increased fetal brain inflammation and cell death are associated with known morbidities in the offspring premature birth (Rees and Inder 2005), and other disorders such as cerebral palsy (Nelson and Grether 1999), autism (Kinney et al. 2008), or schizophrenia (Stefanis et al. 1999).

NMDA receptor antagonists have been extensively reported to reduce brain damage induced by a hypoxic stimulus (Wieloch 1985; Gill et al. 1988; Hagberg et al. 1994). Among these drugs, ketamine is a U.S. Food and Drug Administration–approved noncompetitive NMDA receptor antagonist that has been shown to have anti‐inflammatory properties by reducing levels of IL6 and TNF*α* in early postoperative human patients (Beilin et al. 2007) and animals (Shaked et al. 2004; Lankveld et al. 2005). Previous studies also reported that ketamine has neuroprotective effects in vitro and in animal studies for up to 7 days (Himmelseher and Durieux 2005).

The present study was designed to assess the effect of transient maternal ventilatory hypoxia (i.e., not asphyxia and not prolonged hypoxia) on the fetal cerebral cortex, and the study was designed to test the effect of ketamine on these responses. We approached this question using transcriptomics technology, followed by systems biology analysis to broadly determine the tissue responses to hypoxia and modulation of those responses by ketamine. We chose to investigate the “wide‐angle view” of cellular responses at the mRNA level in order to identify interacting molecular pathways (not to identify single biomarkers) that could lead us to a better understanding of the mechanism of the cellular response to hypoxia and the mechanisms involved in modulation of those responses by ketamine.

## Materials and Methods

These experiments were approved by the University of Florida Animal Care and Use Committee and were performed in accordance with the Guiding Principles for Use of Animals of the American Physiological Society. Sixteen chronically catheterized singleton (*n* = 1) or twin (*n* = 15) ovine fetuses were studied between the gestational age of 122 ± 5 days (full term = 145–147 days).

### Fetal surgery

Ewes were fasted for 24 h before surgery, which was performed on 116 ± 3 days of gestation. Using aseptic technique, fetal and maternal femoral arteries and veins were chronically catheterized as described previously (Chang and Wood 2015). Briefly, the ewes were given 750 mg ampicillin (Polyflex^®^; Boehringer Ingelheim VetMedica, Inc., St. Joseph, MO), then anesthetized and intubated with 0.5–2% isoflurane with oxygen. A set of fetal femoral arterial and venous vascular catheter was surgically placed in the fetal hindlimbs. An amniotic fluid catheter was sutured onto the fetal hindlimb after the vascular catheterization. Before the uterus was sutured closed, 500 mg ampicillin was injected into the amniotic fluid. After catheterization of the fetus(es), catheters were placed into the femoral artery and vein of the ewe, and a nonocclusive catheter was placed in the maternal trachea through the cricoid cartilage as described previously (Chang and Wood 2015). A minimum of 5 days of surgical recovery were allowed before experimentation. Daily postoperative care included monitoring of rectal temperature, administration of antibiotic, and administration of analgesic as needed. All animals, throughout the study, were monitored for anorexia, infection, and other signs of distress.

### In vivo experimental procedures

During all experiments, the ewes were conscious and freestanding in their pens with access to food. The 16 fetuses were randomly assigned to one of the four groups (*n* = 3–5/group): normoxia control, normoxia + ketamine, hypoxia control, and hypoxia + ketamine. In ketamine‐treated groups, ketamine (3 mg/kg) was given intravenously, through the fetal femoral venous catheter, 10 min prior to the hypoxic stimulus (30 min). Hypoxia was induced by infusing nitrogen gas for 30 min directly into the maternal tracheostomy tube as needed to decrease maternal and fetal partial pressures of oxygen (PaO_2_) by approximately 50%. Blood gas responses to the maternal tracheal nitrogen infusion have been reported elsewhere (Chang and Wood 2015). To closely monitor the changes in blood gas compositions (ABL80 Radiometer, Copenhagen, Denmark), both maternal and fetal arterial blood was drawn anaerobically (1 mL) every 10 min. The ewes and fetuses were humanely sacrificed 24 h after the induction of hypoxia or normoxia. Fetal brain was hemisected, with one half subjected to further dissection, snap frozen, and stored at −80°C until future analysis. The other half of the fetal brain was immersion fixed in 4% buffered paraformaldehyde, then processed for paraffin embedding.

### Microarray procedures

Messenger RNA (mRNA) was extracted from fetal frontal cortex using RNeasy Plus Mini Kit (Qiagen, Valencia, CA), with mRNA integrity number (RIN) values between 7.7 and 9.2, were labeled with cyanine 3 CTP with the Quick Amp Labeling Kit (Cat# 5190‐0442; Agilent Technologies, Santa Clara, CA), according to the manufacturer's protocol. The labeled cRNAs ranged from 12.9 to 15.7 pmol Cy3/*μ*g RNA and from 9.4 to 14.4 *μ*g. The cRNA samples were hybridized and processed for one‐channel Sheep Gene Expression Microarray (8 × 15 K slide) – eight arrays with 15,208 oligomers each (Cat# G4813A‐019921; Agilent Technologies) as described earlier (Rabaglino et al. 2012; Wood et al. 2013). The slides were scanned with Microarray Scanner System (G2505‐90021; Agilent) and the measured fluorescence was detected and converted using Agilent Feature Extraction 9.1 software at the Genomics Division of the University of Florida's Interdisciplinary Center for Biotechnology Research. Bioconductor's packages for the R software were employed for raw data import, background correction, data normalization through the quantile method (Smyth and Speed 2003), and statistical analysis of the normalized data. Microarray data have been deposited in the NCBI Gene Expression Omnibus under accession number GSE76110. The functional annotation of gene ontology for significantly up‐ and downregulated genes was analyzed using DAVID Bioinformatics Resources 6.7 (da Huang et al. 2009a, b) and using WEB‐based GEne SeT AnaLysis Toolkit (WebGestalt) (Zhang et al. 2005; Wang et al. 2013). Enrichment analysis was performed using hypergeometric tests, and correction for multiple comparisons was performed according to the method of Benjamini and Hochberg (1995).

### Real‐time PCR

The mRNA used for the microarray was also used for qPCR validations. Complementary DNA (cDNA) was synthesized from mRNA using the Applied Biosystems High‐Capacity Reverse Transcription Kit (Foster City, CA) (Cat# 4368814). The primers used were designed based on the known *Ovis aries* and *Bos taurus* genomes (Table 1) for SYBR green. As a control (housekeeping gene), ovine *β*‐actin primers and probe were used as described previously (Wood et al. 2013). The relative mRNA expression was calculated by the difference in threshold cycle (ΔCt) between the triplicate mean Ct for each gene and the triplicate mean Ct for *β*‐actin mRNA from the same sample.

**Table 1:** Primers for real‐time PCR analyses

Gene	Accession number	Gene name	Forward primer	Reverse primer
AKT	FJ943991.1	V‐Akt murine thymoma viral oncogene homolog 1	TACGGCGCCGAGATCGT	GTCCCTGTACACCACGTTCTTCT
APAF1	NM_001191507.1	Apoptotic peptidase activating factor 1	TTCACTCCAGTGGCCTGTTG	CAAAGCCACCACTGCCAAAT
BAD	NM_001035459.2	BCL2‐associated agonist of cell death	CGATATGGCCGCGAACTC	GCTCTTCGGGCGAGGAA
BAX	XM_003585203.3	BCL2‐associated X protein	TTTCTGACGGCAACTTCAACTG	GGTGCACAGGGCCTTGAG
BCL2	XM_012103831.2	B‐cell CLL/lymphoma 2	GGAGCTGTATGGCCCTAGCA	TGAGCAGTGCCTTCAGAGACA
CASP3	XM_015104559.1	Caspase 3, apoptosis‐related cysteine peptidase	CCATGGTGAAGAAGGAATCATTT	CCCCTCTGAAGAAACTTGCTAATT
CASP8	XM_012142477.2	Caspase 8, apoptosis‐related cysteine peptidase	TGGCTGCCCTCAAGTTCCT	GGAATAGCATCAAGGCATCCTT
CASP9	XM_015099302.1	Caspase 9, apoptosis‐related cysteine peptidase	CAGGTTGCCTCGCTTTGG	CTCGATCATGTCGGGAGTGA
CD14	NM_001077209.1	CD14 molecule	CCTAAAGGACTGCCGACCAA	GCGGCTCCCTGCTTAGCT
CXCL10	NM_001285721.1	Chemokine (C‐X‐C motif) ligand 10	TTGAACTGATTCCTGCAAGTCA	TTCCTTTTCATTGTGGCAATAATCT
CXCL16	XM_015098600.1	Chemokine (C‐X‐C Motif) ligand 16	CACTCCTCTGGAAGGAGCAC	GCTTCTGGTTCTCCCTAGCC
IKBKB	XM_015104530.1	Inhibitor of kappa light polypeptide gene enhancer in B‐cells, kinase beta	GAGACCAGCGGACTGATGGT	CACTCGCACTTTCTTCTCAAAGC
IL1B	EF524261.1	Interleukin 1, beta	CGTGGCCATGGAGAAGCT	GGTCATCATCACGGAAGACATGT
MAP3K14	XM_012149642.2	Mitogen‐activated protein kinase kinase kinase 14	TCACCCCTTCCCATTCCA	TGCTGGTCGTCTACACAGTTTGT
MYD88	NM_001166183.1	Myeloid differentiation primary response 88	GCCTGAGTATTTTGATGCCTTCA	GCTGCCGGATCATCTCATG
NF‐*κ*B1	XM_004020143.3	Nuclear factor of kappa light polypeptide gene enhancer in B‐cells 1	TCCCACAGATGTTCACAAACAGT	GACGCTCAATCTTCATCTTGTGAT
NF‐*κ*BIA	NM_001166184.1	Nuclear factor of kappa light polypeptide gene enhancer in B‐cells inhibitor, alpha	CTACACCTTGCCTGTGAGCA	AGACACGTGTGGCCATTGTA
OAS1	XM_012097881.2	2′‐5′‐Oligoadenylate synthetase 1, 40/46 kDa	GAGGAAAGAGGGCGAGTTCT	GGATGAGGCTCTTCAGCTTG
PKA	NM_001009234.1	Protein kinase, CAMP‐dependent, catalytic, alpha	CCCACGCGCGCTTCTA	TGAGATCAAGCGAGTGCAGGTA
TLR2	XM_012097285.2	Toll‐like receptor 2	GATTCTGCTGGAGCCCATTG	TCATGATCTTCCGCAGCTTACA
TLR4	GU461886.1	Toll‐like receptor 4	ACTCGCTCCGGATCCTAGACT	CCTTGGCAAATTCCGTAGTTCT
TNF	EF446377.1	Tumor necrosis factor	CCCTTCCACCCCCTTGTT	ATGTTGACCTTGGTCTGGTAGGA

### Immunohistochemistry

Fetal brain samples were fixed in 4% buffered paraformaldehyde overnight, and kept in 70% reagent alcohol until embedded with paraffin. Tissue sections (5 *μ*m) were immunostained for ionized calcium‐binding adaptor protein (Iba‐1) antibody (Cat# 019‐19741, Waco Pure Chemical Industries, Richmond, VA; 1:200 dilution), and peroxidase–PAP sandwich technique (Vectastain, Vector Labs, Burlingame, CA). Sections were counterstained with methyl green. Macrophages were quantified in an average area of 181,848 *μ*m^2^ determined in seven images at 40×. Images were randomly made with common areas of the cerebral cortex of all subjects and sections. The total area was calculated using the default color thresholding mechanism of Image J (version 1.48, NIH, Bethesda, MD), where the main parameters (hue, saturation, and brightness) remained constant.

### Calculations and statistical analysis

Data are presented as mean values ± standard error of the mean with consideration for statistical significance at *P *<* *0.05. The ovine Agilent 15.5 K array results were analyzed with Bioconductor's Limma package for R software v.2.15.1 (Smyth 2006), employing moderated *t* test using empirical Bayes method for small sample size per group (*P *<* *0.05). Pairwise comparisons of real‐time PCR data were analyzed using Student's *t* test, with *P *<* *0.05 considered statistically significant.

For the histological analysis, the experiment involved a 2 × 2 factorial completely randomized design with stimulus (hypoxia, normoxia) and treatment (control, ketamine) as factors. A generalized linear model with Poisson distribution for log count data observations was used. Significance was declared at *P *<* *0.05, and if detected, post hoc mean comparison with Bonferroni correction was performed. Least square means and their corresponding standard errors are expressed in the original scale. Statistical analysis was conducted using the Genmod Procedure of SAS/STAT^®^ 9.3 (SAS Institute Inc., Cary, NC).

## Results

### Responses to hypoxia and/or ketamine

**Figure 1:**
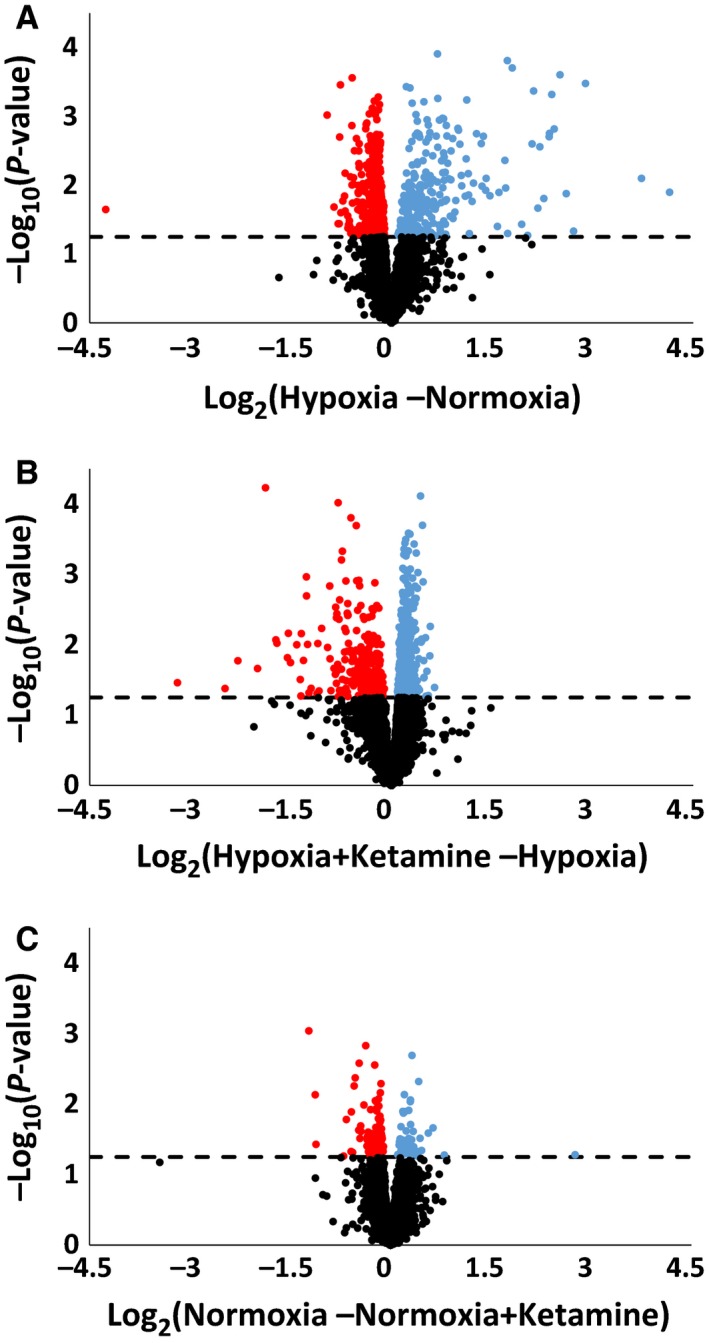
Volcano plot illustrating the relationship of gene expression in fetal frontal cortex measured by log_2_ of fold change. Significantly upregulated (blue) and downregulated (red) genes under hypoxia (A), hypoxia + ketamine (B), and normoxia + ketamine (C). The dotted line indicates whether the gene is statistically significant (above), *P *< 0.05, or not significant (below). Nonsignificant genes are indicated in black dots.

Addition of nitrogen to the maternal inspired gas reduced fetal PaO_2_ to approximately 10 mmHg, and the change in fetal blood gas was not affected by pretreatment with ketamine (Table 2). Cardiovascular and neuroendocrine responses to this stimulus in a larger cohort of fetuses have been reported elsewhere (Chang and Wood 2015). Hypoxia stimulated the upregulation of 248 genes and the downregulation of 480 genes compared to normoxia (Fig. 1). The significantly regulated genes, either up‐ or downregulated, were for analysis of gene ontology, KEGG pathways, common pathways, and associated diseases. Twenty‐four hours after the onset of a 30‐min acute hypoxic stimulus, the biological processes that were upregulated in the fetal frontal cortex were mainly associated with the regulation of immune system response, defense response, response to stress, and response to biotic stimulus (Table 3). The biological processes that were downregulated after the hypoxic insult mainly involved protein metabolic processes, and negative regulation of MAP, ERK1, and ERK2 cascade (Table 3). The enriched KEGG pathway analysis revealed that hypoxia activates the toll‐like receptor and chemokine signaling pathways, cytokine–cytokine receptor interaction, and osteoclast differentiation, and mainly downregulates metabolic pathways (Table 4).

**Table 2:** Fetal blood gas and pH values before (0 min) and at the end (30 min) of maternal ventilatory hypoxia

	0 min	30 min
PaO_2_ (mmHg)		
Nmx	17 ± 1	17 ± 1
Nmx + Ket	17 ± 1	17 ± 1
Hypx	17 ± 1	10 ± 1
Hypx + Ket	17 ± 1	10 ± 1
PaCO_2_ (mmHg)		
Nmx	55 ± 2	56 ± 2
Nmx + Ket	54 ± 2	56 ± 2
Hypx	57 ± 2	53 ± 1
Hypx + Ket	55 ± 2	52 ± 2
pH_a_		
Nmx	7.38 ± .01	7.37 ± .02
Nmx + Ket	7.37 ± .01	7.37 ± .02
Hypx	7.35 ± .01	7.33 ± .02
Hypx + Ket	7.35 ± .01	7.34 ± .02

**Table 3:** Top 10 gene ontology biological processes that were significantly regulated in the frontal cortex

Groups	Biological processes	# Genes involved	Adjusted *P* values
Hypoxia (upregulated compared to normoxia)	Immune response	75	1.06E‐27
	Defense response	75	4.70E‐27
	Immune system process	94	5.61E‐27
	Response to stress	115	2.48E‐23
	Innate immune response	50	4.83E‐23
	Regulation of immune system process	60	1.20E‐21
	Regulation of immune response	46	4.49E‐19
	Positive regulation of immune system process	44	1.46E‐17
	Regulation of defense response	39	1.79E‐17
	Response to biotic stimulus	46	1.79E‐17
Hypoxia (downregulated compared to normoxia)	Cellular protein metabolic process	127	4.00E‐04
	Protein metabolic process	140	2.80E‐03
	Negative regulation of MAP cascade	11	2.80E‐03
	Negative regulation of ERK1 and ERK2 cascade	6	4.30E‐03
	Negative regulation of intracellular protein kinase cascade	13	8.00E‐03
	Water‐soluble vitamin metabolic process	9	1.09E‐02
	Negative regulation of MAP kinase activity	9	1.09E‐02
	Inactivation of MAPK activity	6	1.09E‐02
	Negative regulation of metabolic process	61	1.27E‐02
	Negative regulation of macromolecule metabolic process	58	1.27E‐02
Hypoxia + ketamine (upregulated compared to hypoxia without ketamine)	Organelle organization	97	8.50E‐03
	Cellular protein metabolic process	137	8.50E‐03
	Response to starvation	12	4.29E‐02
	Cellular metabolic process	297	4.29E‐02
	Protein modification by small protein conjugation or removal	37	4.29E‐02
	Cellular response to starvation	10	5.36E‐02
	Macromolecule modification	106	5.36E‐02
	Protein ubiquitination	32	5.36E‐02
	Cellular protein modification process	101	8.24E‐02
	Protein modification by small protein conjugation or removal	32	8.24E‐02
Hypoxia + ketamine (downregulated compared to hypoxia without ketamine)	Defense response	63	7.93E‐17
	Response to stress	104	3.34E‐15
	Regulation of immune response	42	6.46E‐15
	Immune response	58	9.22E‐15
	Regulation of immune system process	51	3.06E‐14
	Response to wounding	58	3.06E‐14
	Regulation of defense response	35	1.67E‐13
	Immune system process	74	1.70E‐13
	Innate immune response	39	1.70E‐13
	Regulation of response to stress	45	3.20E‐13

**Table 4:** Top 10 enriched KEGG pathways that were significantly regulated in the frontal cortex

Groups	Pathway names	# Genes involved	Adjusted *P* values
Hypoxia (upregulated compared to normoxia)	Chagas disease (*American trypanosomiasis*)	13	2.53E‐12
	Complement and coagulation cascades	11	7.75E‐12
	Osteoclast differentiation	13	1.29E‐11
	Toll‐like receptor signaling pathway	12	1.31E‐11
	Cytokine–cytokine receptor interaction	16	4.97E‐11
	*Staphylococcus aureus* infection	9	3.29E‐10
	Pathways in cancer	16	7.88E‐10
	Chemokine signaling pathway	12	9.61E‐09
	Malaria	7	1.38E‐07
	Hematopoietic cell lineage	8	3.13E‐07
Hypoxia (downregulated compared to normoxia)	Vitamin digestion and absorption	5	3.00E‐04
	Metabolic pathways	28	1.30E‐03
	Pathways in cancer	13	1.30E‐03
	TGF‐beta signaling pathway	6	4.50E‐03
	*Vibrio cholerae* infection	5	4.50E‐03
	Wnt signaling pathway	7	1.38E‐02
	Oocyte meiosis	6	1.39E‐02
	Amino sugar and nucleotide sugar metabolism	4	1.59E‐02
	Cell cycle	6	1.75E‐02
	Collecting duct acid secretion	3	2.10E‐02
Hypoxia + ketamine (upregulated compared to hypoxia without ketamine)	Metabolic pathways	46	4.97E‐10
	mRNA surveillance pathway	9	6.34E‐05
	mTOR signaling pathway	7	2.00E‐04
	Tight junction	9	9.00E‐04
	Glycine, serine, and threonine metabolism	5	9.00E‐04
	Spliceosome	9	9.00E‐04
	Insulin signaling pathway	9	1.20E‐03
	TGF‐beta signaling pathway	7	1.40E‐03
	Huntington's disease	10	1.40E‐03
	Vitamin digestion and absorption	4	1.90E‐03
Hypoxia + ketamine (downregulated compared to hypoxia without ketamine)	Complement and coagulation cascades	12	4.74E‐13
	Chagas disease (*American trypanosomiasis*)	13	1.50E‐12
	*Staphylococcus aureus* infection	10	1.88E‐11
	Malaria	9	2.17E‐10
	Osteoclast differentiation	12	2.17E‐10
	Pathways in cancer	15	1.07E‐08
	Leishmaniasis	8	8.78E‐08
	Toll‐like receptor signaling pathway	9	8.78E‐08
	Chemokine signaling pathway	11	1.24E‐07
	Amebiasis	8	1.50E‐06

Ketamine had a small effect on its own. In the fetuses that were treated with ketamine under normoxic conditions (Fig. 1C), only 50 genes were significantly upregulated compared to normoxia control, and these did not provide any significant biological processes or pathways. However, there were 105 genes that were downregulated by ketamine and the significant biological processes included cellular potassium ion transmembrane transport (three genes, adjusted *P *=* *3.78E‐2), mitotic prometaphase (five genes, adjusted *P *=* *3.53E‐2), viral reproduction (14 genes, adjusted *P *=* *2.15E‐2), and interspecies interaction between organisms (10 genes, adjusted *P *=* *3.53E‐2).

Ketamine significantly upregulated 570 genes and downregulated 250 genes during hypoxia (compared to hypoxia without ketamine pretreatment) (Fig. 1B). Biological process terms significantly associated with the upregulated genes were biological process terms organelle organization, cellular protein metabolic process, and macromolecule modification (Table 3). Significantly associated biological process terms with downregulated genes were defense response, response to stress, and regulation of immune response (Table 3). KEGG analysis revealed that ketamine treatment before hypoxia upregulated metabolic pathways and downregulated toll‐like receptor and chemokine signaling pathways (Table 4).

### Modulation of responses to hypoxia by ketamine

**Figure 2:**
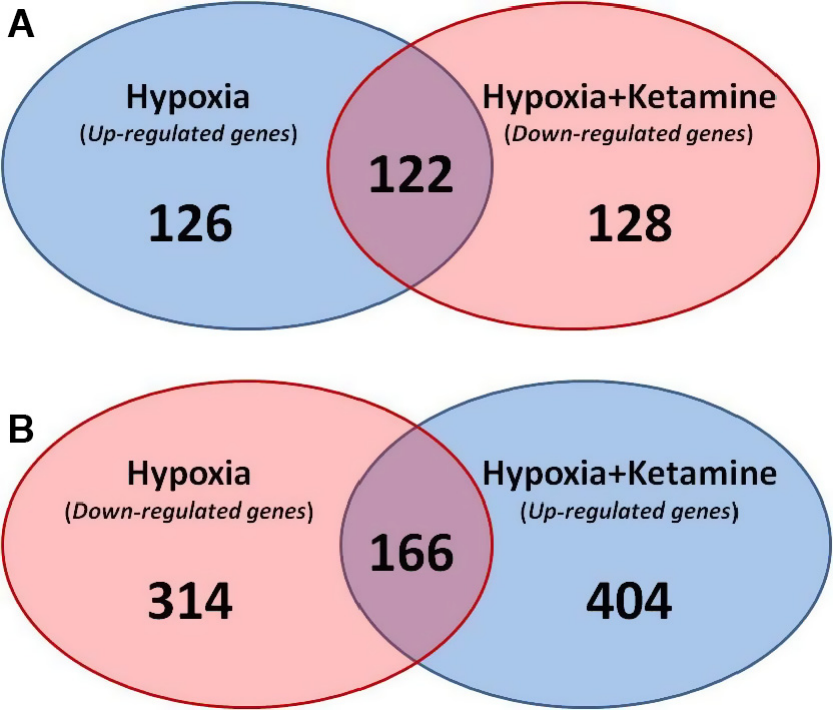
Venn diagram of the number of genes that were significantly regulated by hypoxia and hypoxia + ketamine. (A) Venn diagram showing the number of significant genes that were upregulated by hypoxia (blue), downregulated with ketamine (red), and the common genes involved in both groups (purple). (B) Venn diagram of significant genes that was downregulated by hypoxia (red), upregulated with ketamine (blue), and the common genes involved in both groups (purple).

To determine the modulatory effect of ketamine pretreatment on hypoxia, the genes that were significantly upregulated by hypoxia (vs. normoxia) were compared with the genes that were significantly downregulated by hypoxia + ketamine (vs. hypoxia alone) (Fig. 2A). There were 126 genes that were uniquely upregulated by hypoxia, 128 genes that were downregulated by hypoxia + ketamine, and 122 common genes between the both groups. Gene ontology analysis revealed that these common genes were significantly associated with biological process terms related to the regulation of immune, defense, and stress response (Table 5). The enriched KEGG pathway analysis indicated the involvement of toll‐like receptor signaling, chemokine signaling, and cytokine–cytokine receptor interaction. The major common pathways associated with these 122 common genes were immune, inflammatory, apoptosis, metabolic, and cellular division (Table 6). The analysis of enriched disease‐associated genes yielded statistically significant association with the following terms: inflammation, necrosis, immune system, and autoimmune diseases (Table 7).

**Table 5:** Top 10 gene ontology biological processes and enriched KEGG pathways that were significantly upregulated in the frontal cortex during acute hypoxic stress and downregulated by ketamine treatment before hypoxia

Analysis	Pathways, processes, functions, components	# Genes involved	Adjusted *P* values
Biological process	Immune response	42	7.39E‐17
	Immune system process	53	7.39E‐17
	Defense response	43	7.39E‐17
	Regulation of immune system process	38	1.12E‐16
	Regulation of immune response	30	4.30E‐15
	Response to wounding	40	6.90E‐15
	Positive regulation of immune system process	29	2.09E‐14
	Response to stress	63	2.09E‐14
	Innate immune response	28	9.60E‐14
	Response to lipid	28	6.08E‐13
Molecular function	Protein binding	80	5.78E‐07
	Cytokine receptor binding	10	2.00E‐04
	Interleukin‐6 receptor activity	2	1.30E‐03
	Receptor binding	22	1.30E‐03
	Chemokine receptor binding	5	1.30E‐03
	Interleukin‐6 binding	2	1.30E‐03
	Cell surface binding	5	1.30E‐03
	Enzyme inhibitor activity	10	1.50E‐03
	Lipoteichoic acid binding	2	1.70E‐03
	Peptidase regulator activity	8	1.70E‐03
KEGG pathways	Chagas disease (*American trypanosomiasis*)	11	2.78E‐13
	Complement and coagulation cascades	10	2.78E‐13
	*Staphylococcus aureus* infection	9	8.98E‐13
	Pathways in cancer	12	2.32E‐09
	Toll‐like receptor signaling pathway	8	6.40E‐09
	Osteoclast differentiation	8	3.27E‐08
	Cytokine–cytokine receptor interaction	10	3.79E‐08
	Malaria	6	5.32E‐08
	Chemokine signaling pathway	8	4.61E‐07
	Systemic lupus erythematosus	7	7.49E‐07

**Table 6:** Top 10 common pathways associated with the genes that were significantly upregulated in the frontal cortex in response to hypoxia and downregulated by ketamine treatment before hypoxia

Common pathways	# Genes involved	Adjusted *P* values
Immune system	27	6.57E‐24
Innate immune system	17	6.54E‐19
Integrin family cell surface interactions	32	7.15E‐19
TRAIL signaling pathway	31	2.09E‐18
Glypican 1 network	30	1.01E‐17
Signaling events mediated by hepatocyte growth factor receptor (c‐Met)	29	1.59E‐17
Insulin pathway	29	1.59E‐17
S1P1 pathway	29	1.59E‐17
Arf6 downstream pathway	29	1.59E‐17
Arf6 signaling events	29	1.59E‐17

**Table 7:** Top 10 enriched diseases associated with the genes that were significantly upregulated in the frontal cortex during acute hypoxic stress, but was downregulated with ketamine

Diseases	# Genes involved	Adjusted *P* values
Inflammation	26	2.00E‐24
Necrosis	21	2.44E‐20
Immune system diseases	26	5.81E‐20
Autoimmune diseases	20	4.53E‐17
Chorioamnionitis	15	1.23E‐16
Infection	20	2.16E‐15
Preterm rupture of membranes	13	7.23E‐14
Bronchiolitis	14	1.21E‐13
Virus diseases	18	1.50E‐13
Bacterial infections	13	1.64E‐13

Comparison of the 166 common genes between the downregulated hypoxia versus the upregulated hypoxia + ketamine (Fig. 2B) revealed only one significant gene ontology biological process: cellular protein metabolic process (50 genes, adjusted *P *=* *3.34E‐2). The KEGG analysis pathway yielded a few significant pathways, with metabolic pathways as the main outcome (nine genes of the 166 common genes). The rest of the pathways involved very few genes, which varied between 1 and 3% (2–5 genes of the 166 common genes).

### Inflammation and immune responses

**Figure 3:**
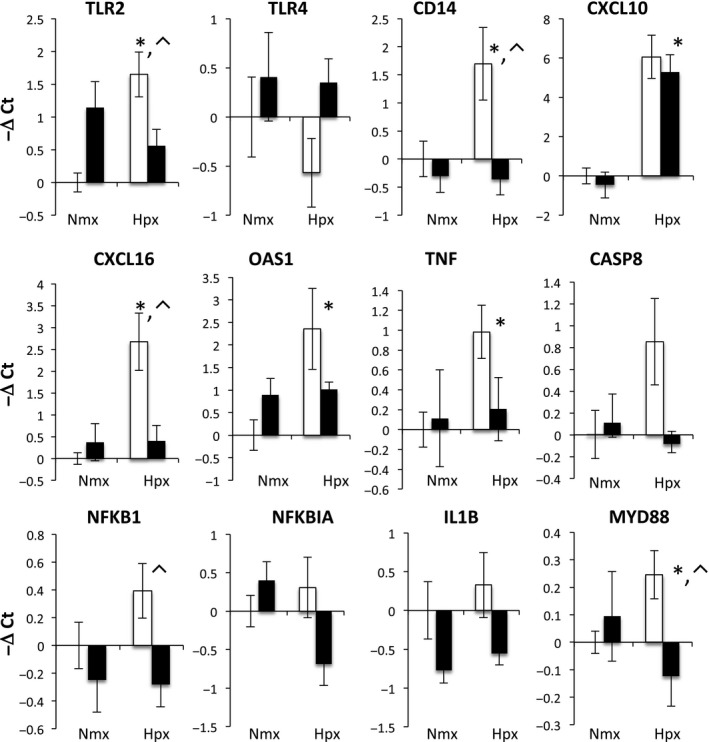
Gene expression (mRNA abundance) was measured by real‐time qPCR for genes in the toll‐like receptor inflammation pathway. Values are represented as delta cycle threshold (ΔCt) compared to the normoxia control (no hypoxia and no ketamine) group. Open bars represent experiments in which ketamine was not administered. Filled bars represent experiments in which ketamine was administered before normoxia or hypoxia. The criterion for statistical significance was *P *<* *0.05 (Student's *t* test). Data are presented as means ± SEM, and the *y*‐axis scale varies between plots. *Statistically significant difference in hypoxia + ketamine group compared to normoxia control. ⌃Statistically significant difference in hypoxia control group compared to hypoxia + ketamine group.

**Figure 4:**
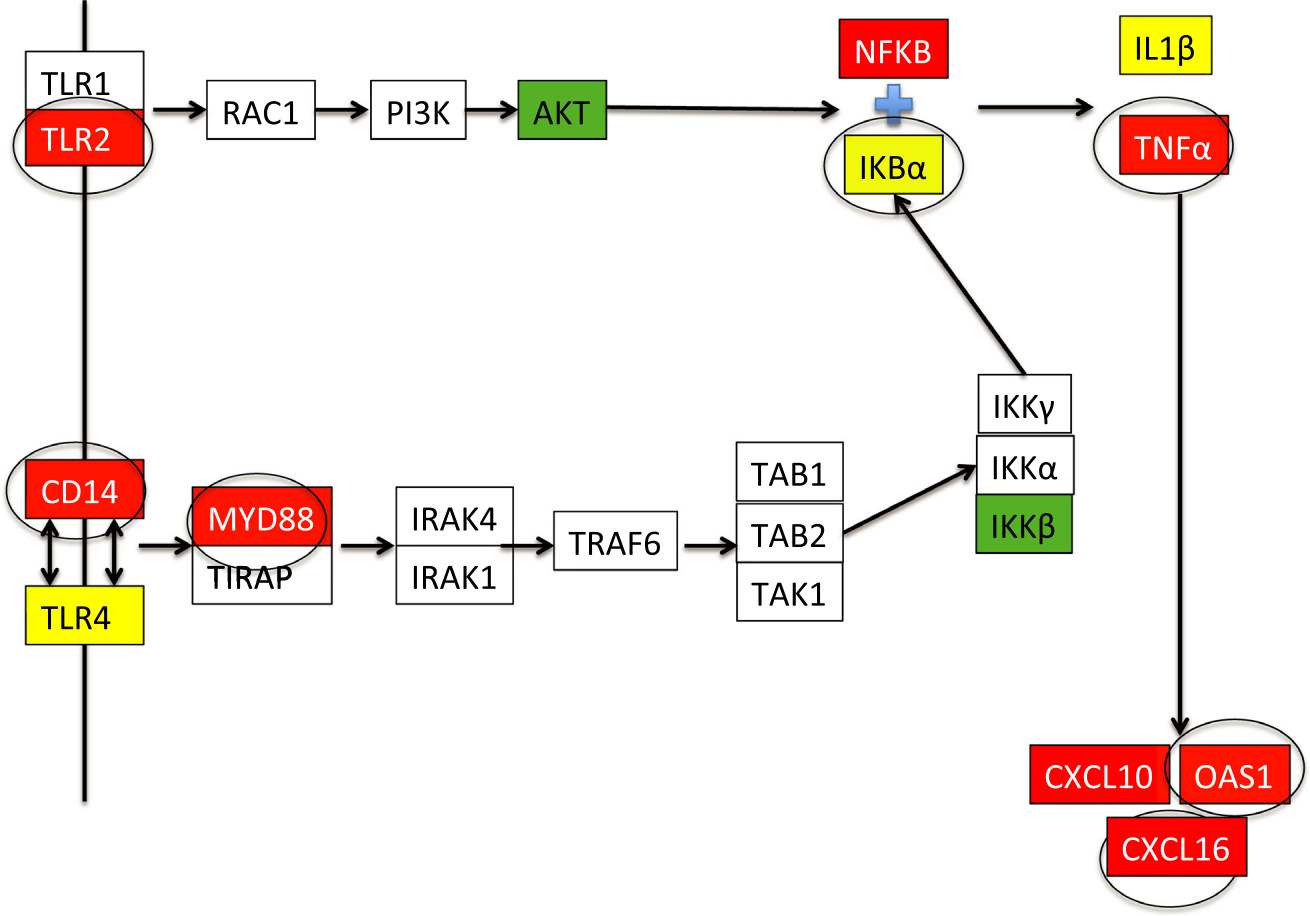
Graphical representation of the toll‐like receptor inflammation pathways queried by qPCR in the present studies. Boxes that are uncolored were not tested by qPCR. Red color denotes genes whose expression was increased by hypoxia. Green color denotes genes whose expression as decreased by hypoxia. Yellow color denotes genes whose expression was not changed by hypoxia. Circled boxes represent genes whose response to hypoxia was significantly altered by ketamine.

**Figure 5:**
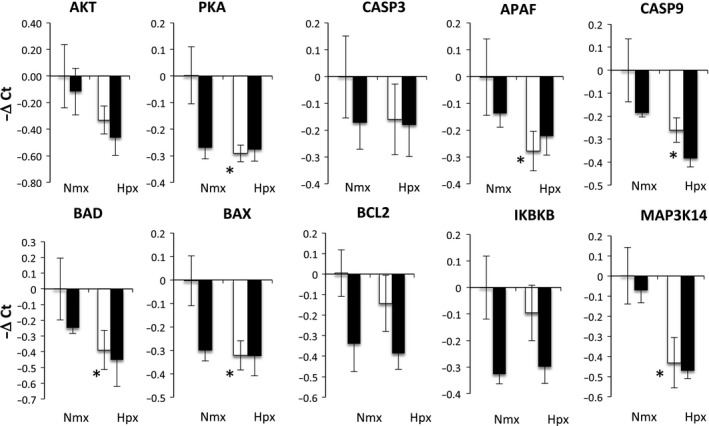
Gene expression (mRNA abundance) was measured by real‐time qPCR for genes related to apoptosis. Values are represented as delta cycle threshold (ΔCt) compared to the normoxia control (no hypoxia and no ketamine) group. Open bars represent experiments in which ketamine was not administered. Filled bars represent experiments in which ketamine was administered before normoxia or hypoxia. The criterion for statistical significance was *P *<* *0.05 (Student's *t* test). Data are presented as means ± SEM, and the *y*‐axis scale varies between plots. *Statistically significant difference of hypoxia + ketamine group compared to normoxia control.

The transcriptomics analysis predicts that hypoxia increases inflammation, and that ketamine ameliorates this response. To explore this possibility, we performed qPCR on genes within the inflammation pathway (not all of these genes were represented by the microarray probes). Hypoxia stimulated increases in many genes within the TLR‐mediated inflammatory cascade: TLR2, CD14, MYD88, NF‐*κ*B1, TNF*α*, CXCL110, CXCL16, and OAS1 (Fig. 3). Ketamine prior to hypoxia altered the expression of TLR2, CD14, MYD88, CASP8, IKBA, TNF*α*, OAS1, and CXCL16 compared to hypoxia alone. Shown in Figure 4 is a modified flow chart of the TLR2‐ and TLR4‐dependent inflammatory cascade, illustrating that the inflammatory response to hypoxia and the modification of the response by ketamine appears to involve the entire cascade. Interestingly, hypoxia appeared to reduce the expression of genes involved in apoptosis, and ketamine appeared to have no modulatory effect on these genes (Fig. 5).

**Figure 6:**
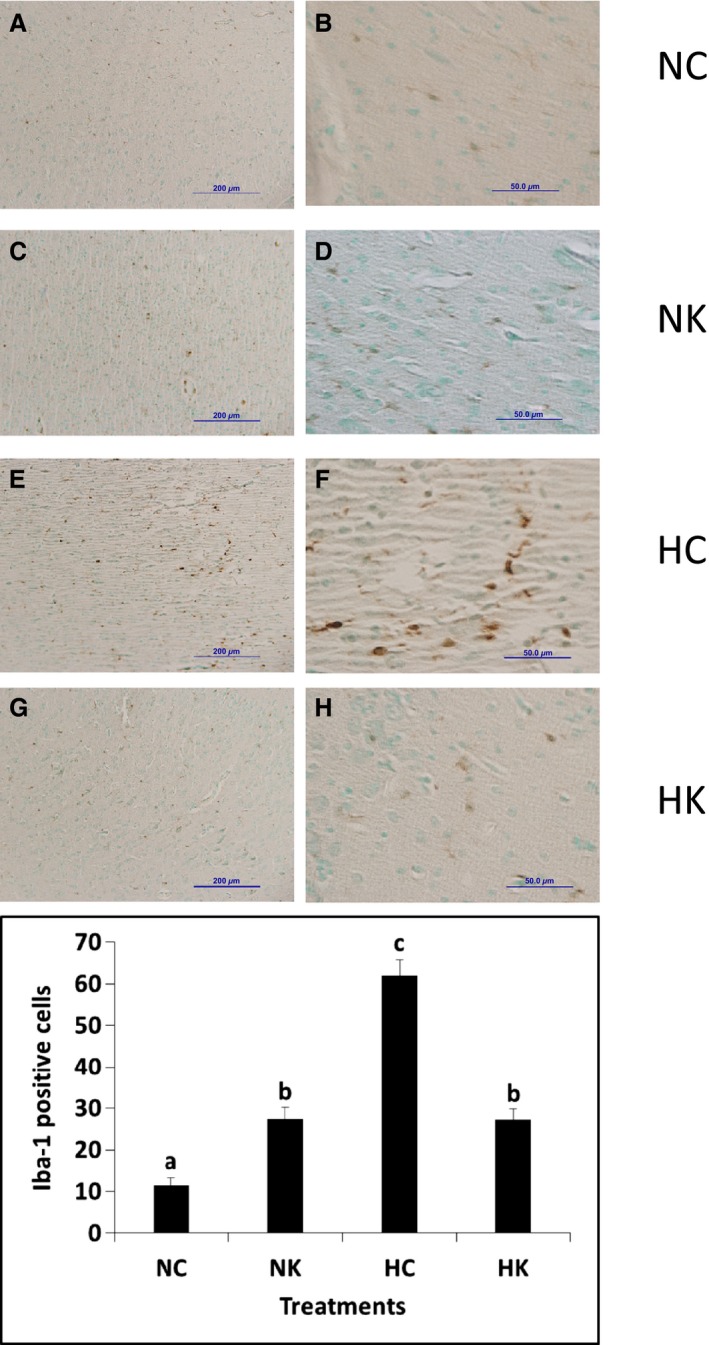
Immunohistochemical analysis of cells expressing Iba‐1 immunoreactivity in the frontal cerebral cortex. Representative images at low (A, C, E, G) and high (B, D, F, H) magnification from animals exposed to normoxia with no ketamine pretreatment (A, B), animals exposed to normoxia following ketamine pretreatment (C, D), animals exposed to hypoxia with no ketamine pretreatment (E, F), and animals exposed to hypoxia following ketamine pretreatment (G, H) fetuses. Sections were counterstained with methyl green. Macrophage counts per field at 40× in the cerebral cortex (graph) from seven images per animal and three to four animals per group. Data are expressed as means ± SEM. Different letters indicate statistically significant difference (*P *<* *0.05). Scale bars 200 *μ*m (A, C, E, G), 50 *μ*m (B, D, F, H). NC, normoxia control; NK, normoxia + ketamine; HC, hypoxia control; HK, hypoxia + ketamine.

Together, the transcriptomics and qPCR suggest that there is an immune response within the fetal cerebral cortex that is reduced by ketamine. To validate this conclusion, we performed immunohistochemistry on cerebral cortex, staining for Iba‐1 (a macrophage/microglia marker to test for accumulation of macrophages or microglia). The density of Iba‐1‐positive cells was significantly increased after hypoxia; this response was attenuated by ketamine (Fig. 6). Ketamine alone did not have a statistically significant effect on the number of Iba‐1‐positive cells (Fig. 6). The abundance of Iba‐1‐positive cells in the cerebral cortex mirrored the transcriptomics response within the inflammatory cascade (hypoxia increases the number of positive cells and ketamine reduces the response).

## Discussion

The present study represents a marriage of two technologies: whole animal physiology and transcriptomics‐based systems biological modeling and analysis of the physiology. We have been successful, for example, in modeling and validating changes in cardiac physiology in the fetus exposed to chronic elevations in maternal cortisol (Richards et al. 2014). In the present study, systems modeling of transcriptomics in the frontal cerebral cortex 24 h after hypoxia indicate that there are increased inflammation and immune responses as well as the anticipated decreased cellular and protein metabolic processes. The immune response predicted by the model was validated using histological evidence of increased macrophage/microglia number in the fetal cerebral cortex. Administration of a standard clinical dose of ketamine (based on estimated fetal body weight) prior to hypoxia modified and, in some aspects, prevented or greatly reduced these responses, suggesting that ketamine may be useful for its anti‐inflammatory action in the fetal brain. The present study, because it focuses on pathways activated by hypoxia and inhibited by ketamine, is limited to understanding tissue responses and not long‐term outcome. While the pathway analysis reveals heretofore unknown responses to what would be considered a relatively common fetal stressor, it does not specifically address demyelination, cell death, or either short‐ or long‐term deficits in cortical function.

We designed the present study to test the hypothesis that ketamine, a noncompetitive NMDA receptor antagonist, attenuates the inflammation that results from hypoxia. In order to identify the effect of ketamine on the hypoxic fetal frontal cortex, we examined the common genes that were both significantly upregulated by hypoxia and significantly downregulated by ketamine pretreatment before the hypoxia (Fig. 2). The results indicated that inflammation pathways were significantly upregulated by hypoxia, and that cellular and protein metabolic processes were downregulated. The effects of ketamine opposed those of hypoxia are upregulation of metabolic processes and downregulation of inflammatory pathways (Tables 3 and 4).

Neonatal and perinatal brain inflammation is characterized by increased cytokines, including IL1, IL6, and TNF*α* (Szaflarski et al. 1995; Hagberg et al. 1996; Cieslak et al. 2013). These proinflammatory cytokines, along with the transcription factor NF‐*κ*B, one of the main regulators of the inflammatory response, have been reported to have a direct cytotoxic effect on neurons and glial cells (Volpe 2001). Inflammatory mediators promote the recruitment, activation, and infiltration of immune cells in the brain, and contribute to the severity of the hypoxic injury (Castillo‐Melendez et al. 2013; Jellema et al. 2013). Our transcriptomics model and the immunohistochemical validation of the model suggest that the response to hypoxia involves alterations in the abundance of macrophages or microglia within the cerebral cortex. Unknown is the nature of the stimulus to the increase in macrophage/microglia abundance. It could be directly related to the cellular hypoxia and energy starvation, or possibly a secondary response (e.g., secondary to changes in neurotransmission in chemoreceptor‐sensitive pathways in the fetal brain). We have previously reported that, 1 h after hypoxia, transcriptomics modeling indicated a reduction in cellular metabolism and the beginning of an inflammatory/immune response in the fetal hypothalamus (Wood et al. 2014). We do not know whether 30 min of moderate hypoxia (reduction in fetal PaO_2_ of approximately 50%) actually damages the neurons and glia in the fetal brain. Physiologically, the fetal brain reduces oxygen demand in parallel with the reduction in oxygen delivery (Jones et al. 1983; Rurak et al. 1990), likely affording some protection from damage. Nevertheless, it is possible that reoxygenation is a direct insult to the brain, increasing the formation of oxygen radicals and other proinflammatory stimuli (Maulik et al. 1999). However, it is also possible that there are other events that stimulate inflammation in the fetal brain and other tissues (i.e., release of proinflammatory substances from the placenta or other tissues). We find it interesting and intriguing that the increase in macrophages/microglia in the fetal cerebral cortex appears to be very similar to an increase in macrophages in the renal cortex of the fetus, recently reported elsewhere (Chang et al. 2016).

We were surprised to see the magnitude of the increase in abundance of macrophages or microglia in the fetal cerebral cortex after hypoxia, but we were not surprised to find an active involvement of the fetal immune system in the response. In other experiments, we have evidence of immune development within the fetal brain in the latter half of gestation (Rabaglino et al. 2014). Interestingly, the consensus trend in gene expression in the latter half of gestation in the fetal sheep reflects pathways involved in hematopoietic immune cell development (Rabaglino et al. 2014). We do not know whether the Iba‐1‐positive cells observed in the present experiments were microglia that were already present in the fetal brain or whether they were macrophages that entered into the fetal cerebral cortex from the blood. We suspect the latter because we have observed what appear to be Iba‐1‐positive cells migrating between blood and brain parenchyma (Fig. 6).

In addition to testing inflammation pathways by qPCR, we tested gene expression in pathways related to apoptosis. Calcium influx into the neurons promotes neuronal apoptosis mainly via the caspase signaling pathway (Vannucci and Hagberg 2004). Several studies have reported that hypoxic stimuli elevate concentrations of the apoptotic factors caspase 3, 7, 8, and 9 (Northington et al. 2001; Zhu et al. 2003; Vannucci and Hagberg 2004), and other adaptor proteins such as BAX/BCL2 (Cheng et al. 2003), MYD88 (Wang et al. 2009), and NF‐*κ*B (Liu et al. 2005). While we expected there to be an increase in expression of genes mediating apoptosis 24 h after hypoxia, we found that many of these genes were downregulated (AKT, CASP3, CASP9, BAD, BAX, BCL2) and not further altered by ketamine (Fig. 5). Exceptions to this trend were MYD88 and CASP8, which were upregulated by hypoxia and blunted by ketamine (Fig. 3). Based on these results, we conclude that hypoxia results in alterations in apoptosis as well as inflammation, but that the effects on apoptosis appear to be favoring reduction of apoptosis, possibly because apoptosis is an energy requiring process. Apoptosis is an integral aspect of brain development (synaptogenesis and neuronal pruning) in the fetus, and it is not clear whether a transient alteration in the rate of apoptosis could permanently alter the course of cortical development.

Ketamine is known to bind an array of receptors besides NMDA receptors including dopamine receptors (Kapur and Seeman 2002), non‐NMDA glutamate receptors, opioid receptors, nicotinic and muscarinic cholinergic receptors (Kohrs and Durieux 1998), serotonin receptors (Yamakura et al. 2000; Kapur and Seeman 2002), and weakly influencing the glycine and GABA_A_ receptors (Yamakura et al. 2000). Nevertheless, we do not know whether the anti‐inflammatory action of ketamine in this study is related to a neuropharmacologic action of the drug. Other studies have also supported the role of ketamine as an anti‐inflammatory agent when used in both clinically (Yli‐Hankala et al. 1992; Beilin et al. 2007; Welters et al. 2011) and experimentally (Koga et al. 1994; Takenaka et al. 1994; Kawasaki et al. 1999). Investigators have reported that ketamine reduces the levels of TNF*α*, IL1*β*, IL6, and IL8, which are the main innate immune system modulators (Larsen et al. 1998; Kawasaki et al. 1999, 2001; Shaked et al. 2004; Lankveld et al. 2005; Gurfinkel et al. 2006). For example, it is possible that in the present experiments ketamine has a direct effect on macrophages to reduce the secretion of cytokines and other inflammatory mediators. Tan et al. (2015) have reported that ketamine has a direct effect on macrophages to reduce NF‐*κ*B‐mediated responses to lipopolysaccharide. Indeed, such an action of ketamine would be consistent with our results.

In summary, we have used a combined approach of whole animal physiology and transcriptomics‐based systems biology modeling to explore the response of the fetal cerebral cortex to transient hypoxia in the late gestation fetal sheep. Our results are in agreement with previous results from other laboratories that indicate an inflammation response, but we have provided evidence of anti‐inflammatory actions of clinically relevant doses of ketamine, and we suggest that ketamine might be useful for reducing (fetal or neonatal) cortical damage after transient hypoxia or ischemia. The results indicate an inflammation response that involves the entire toll‐like receptor inflammation pathway with influx of microglia or macrophages into the cerebral cortex. We conclude that the inflammation response in the cerebral cortex is similar to that in the kidney cortex, suggesting that the ultimate stimulus to the inflammation might be secondary to signaling that originates in other fetal or placental tissues.

## Perspectives

Thirty minutes of maternal ventilatory hypoxia of a magnitude expected upon acute exposure to 12,000 feet altitude (Rahn and Otis 1949) results in fetal brain inflammation and accumulation of microglia or macrophages in the fetal cerebral cortex. The vigorous nature of this response is surprising to us, and it is reminiscent of inflammation in the fetal renal cortex after the same hypoxic stimulus (Chang et al. 2016). Will milder hypoxia (either in magnitude or duration) have a similar effect? Perhaps more important than the questions of cause or sensitivity is the question of whether this inflammatory response demarks tissue damage or whether it plays a positive role in fetal brain development. Transient hypoxia can be caused, for example, by air travel in pressurized aircraft (Hampson et al. 2013), by visits to high altitude (Rahn and Otis 1949), or even perhaps by sleep apnea in late gestation (Venkata and Venkateshiah 2009). How common are bouts of fetal brain inflammation in the normal fetus? Are these somehow causally related to hematopoietic immune cell development in the fetal brain (Rabaglino et al. 2014)? Does transient hypoxia have negative or even positive long‐term consequences?

## Conflict of Interest

None declared.
